# Application of resistance trainer for dynamic mechanical properties of conjugated polymer materials in daily physical strength tensile training

**DOI:** 10.3389/fchem.2023.1282908

**Published:** 2023-10-16

**Authors:** Wei Hu

**Affiliations:** School of Physical Education, Chongqing Technology and Business University, Chongqing, China

**Keywords:** conjugated polymer materials, dynamic mechanical properties, resistance trainers, daily physical strength training, application prospects

## Abstract

Resistance training is an important means to improve human function and health level, and tensile training, as an important form, has significant effects on enhancing muscle strength, improving body flexibility, and promoting body coordination. Tensile training can provide external resistance through the use of resistance trainers, allowing muscle groups to work under load. This load stimulation can gradually promote muscle adaptation and enhancement. This article took conjugated polymer materials as the research object. By analyzing and testing their dynamic mechanical properties, the mechanical response characteristics of polyacetylene materials were analyzed using strain rate functions. The mechanical properties and deformation behavior of polyacetylene materials under different conditions were evaluated. It was found that conjugated polymer materials had good strength, elasticity, and wear resistance, making them suitable for making resistance trainers. The experimental results indicated that this study used a conjugated polymer material resistance trainer to train the testing personnel for a period of 3 months. During this training period, the tester’s endurance increased by 21%–29%, flexibility increased by 17%–25%, and explosive power increased by 24%–31%. The research results indicated that conjugated polymer materials had good plasticity and adjustability in the preparation of resistance training devices. Resistance devices of different shapes, sizes, and intensities can be prepared according to the needs and purposes of trainers, providing diverse and personalized choices for daily physical tensile training. The conjugated polymer material resistance trainer has significant effects and can effectively improve the physical fitness of testers. In addition, conjugated polymer materials exhibit excellent tensile performance and durability in resistance training, which can effectively meet the needs and requirements of trainers.

## 1 Introduction

With the continuous improvement of people’s awareness of healthy living, physical exercise has become an indispensable part of daily life. Among them, tensile training is a widely used training method in the fields of sports and fitness. By utilizing external resistance devices, the muscles of various parts of the body are trained and exercised to improve their strength, endurance, and sensitivity. At present, the materials used for resistance training equipment are mostly traditional materials such as metal and plastic, which have limited their widespread application and promotion due to their high preparation cost, large weight, and large volume. Therefore, finding a new type of material that can prepare high-performance, lightweight, and small volume resistance devices has important application value and significance. Conjugated polymer materials, as a new type of high-performance material, have been widely used in fields such as electronics, optoelectronics, and energy due to their unique electronic structure and conductivity. Meanwhile, conjugated polymer materials have excellent mechanical properties and durability, making them a promising resistance training material. By adjusting the structure and morphology of conjugated polymer materials, training devices with different strengths and resistance levels can be prepared. These devices can adapt to different training needs, including strength training, endurance training, and flexibility training. One of the advantages of conjugated polymer materials as resistance training materials is their good plasticity and customizability. These materials can adjust their mechanical properties by changing their chemical composition, polymerization method, and structural design to meet specific training requirements. In recent years, researchers have made significant progress in the preparation and application of conjugated polymer materials. However, there is still relatively little research on the application of conjugated polymer materials in the field of resistance training, and further in-depth exploration is needed to explore their application effects and advantages in resistance training.

Resistance trainers have a variety of applications in daily physical tensile training and are suitable for individualizing training for different training goals and needs. The research by Weakley, Jonathon J.S. aimed to explore the relationship between training practices, physical measurements, and physiological changes of young rugby players during the 12 weeks season. The results showed significant differences in training practices among young rugby players, and overall improvements in physical measurements and physiological characteristics were observed during the 12 weeks season. In addition, the load capacity of resistance training and the number of composite movements are closely related to changes in physical characteristics (Weakley Jonathaon et al., 2019). Shinichiro Morishita described the RPE (Rating of Perceived Exercise) scale for resistance training conducted by elderly people. RPE is related to the intensity of resistance training, and the RPE scale is used to determine the intensity of resistance training in elderly people or patients. The RPE scale can determine the intensity of resistance training for elderly people, and if they do not have a specific resistance training machine, they can be used at home or in nursing homes. RPE is simple and useful, which can improve skeletal muscle strength or other health related issues (Morishita et al., 2019). Farzane Saeidifard believed that the benefits of aerobic exercise had been well studied. There is currently no consensus on the relationship between resistance training and major cardiovascular adverse events. He also searched for randomized trials and cohort studies to evaluate the relationship between resistance training and mortality and cardiovascular events (Saeidifard et al., 2019). Li Yi developed an active upper limb exoskeleton strength training device for restoring patients’ upper limb muscle strength training and motor control ability training (Yi and Yu, 2019). The above scholars believe that resistance trainers have significant effects in improving muscle strength, endurance, explosive power, flexibility, and joint stability.

The selection of appropriate materials has a direct impact on the performance and effectiveness of resistance trainers. Guo Xiaozheng believed that liquid crystal engineering plastics and liquid crystal fibers can be used to make rocket engine shells, bulletproof vests, high-end tires, *etc.* Liquid crystal polymer materials are developing into fields such as home appliances, medical devices, and sports equipment, all of which indicate the broad development prospects of liquid crystal materials (Guo et al., 2022). Yang Lei believed that fiber composite materials under high-tech technology have been widely used in sports equipment due to their significant characteristics of light weight and high strength (Yang, 2019). Wang Linjian believed that carbon fiber reinforced composite materials are widely used in aerospace, transportation, sports equipment, and other fields due to their high strength, high temperature resistance, radiation resistance, and chemical corrosion resistance (Wang et al., 2019). Li Haipeng believed that with the continuous development of technology, sports training is also developing towards new ideas, new technologies, new equipment, and other aspects. The application of wearable devices based on mobile internet and cloud platforms with wireless transmission and fast real-time feedback functions in sports training is becoming increasingly widespread and in-depth, providing a more convenient and scientific way to explore the biological significance of sports training (Li et al., 2020). The above scholars believe that high-quality materials can provide moderate elasticity, durability, adjustability, and safety to ensure that resistance trainers can meet different training objectives and personal needs, and maintain stable quality and effectiveness in long-term use.

This article took conjugated polymer materials as the research object, and explored their application effects and advantages in the field of resistance training by analyzing and testing their dynamic mechanical properties. By testing the strain rate function, the mechanical response characteristics of conjugated polymer materials were understood. The research results indicated that conjugated polymer materials have good plasticity and adjustability, and have important advantages in the preparation of resistance devices. In addition, conjugated polymer materials exhibit excellent tensile performance and durability in resistance training, which can effectively meet the needs and requirements of trainers. This article believed that the resistance trainer for dynamic mechanical properties of conjugated polymer materials has broad application prospects and potential in daily physical strength tensile training, and is worth further research and development.

## 2 Design method of resistance trainer based on conjugated polymer materials

### 2.1 Potential of conjugated polymer materials in the field of resistance training

Conjugated polymer materials are a type of polymer material with a special structure, which has high conductivity and thermal conductivity. Due to its unique structure, conjugated polymer materials exhibit some unique performance characteristics, making them potential in the field of resistance training.

Conjugated polymer materials have excellent conductivity. Its intramolecular conjugated structure allows electrons to conduct freely within the molecule, resulting in a high conductivity of the material. This means that conjugated polymer materials can be used as electrode materials to manufacture training devices with current conduction functions, such as resistors and resistance devices. These devices can provide precise current regulation and resistance control, thereby achieving more refined resistance training.

Conjugated polymer materials have good controllability and plasticity. By controlling the molecular structure and adding different dopants, the conductivity and mechanical properties of conjugated polymer materials can be adjusted to meet different resistance training needs. At the same time, conjugated polymer materials can change their conductivity and morphological structure by adjusting external conditions such as electric field, temperature, and light, thereby achieving precise regulation of resistance training. This regulation can provide more flexible and personalized choices for resistance training to meet specific training goals and requirements.

Conjugated polymer materials also have good durability and renewability. Conjugated polymer materials usually have high thermal and chemical stability, and can withstand high temperatures and chemical corrosion conditions, which enables them to be stably applied in the field of resistance training for a long time. In addition, conjugated polymer materials can be regenerated and reused through controllable synthesis methods and recycling technologies, reducing their impact on the environment and improving resource utilization efficiency.

Resistance training is an important aspect of maintaining skeletal muscle mass and muscle strength (Colquhoun Ryan et al., 2018; Aamann et al., 2020). Based on the structural and performance characteristics of conjugated polymer materials, they have broad application potential in the field of resistance training. For example, conjugated polymer materials can be used to manufacture high-performance resistance devices and resistors for training the strength and endurance of different muscle groups. It can be used to manufacture intelligent resistance training equipment, achieving personalized resistance training schemes through precise current and resistance adjustment. It can also be used to manufacture flexible resistance bands and resistance clothing, providing a comfortable training experience and a comprehensive resistance training effect. Obesity and strength are two common long-term goals of resistance training, which are regulated by manipulating many variables, including load, volume, exercise sequence, exercise selection, and inter group rest intervals (Wilk et al., 2021).

Conjugated polymer materials are a highly promising resistance training material, and their structure and performance characteristics provide new possibilities for manufacturing high-performance, intelligent, and sustainable resistance training equipment. With the continuous improvement of the requirements for resistance training effectiveness and comfort, the application prospects of conjugated polymer materials in the field of resistance training would be more broad.

### 2.2 Design principle and preparation method of resistance trainer

(1) Selection and blending of conjugated polymer materials

When selecting conjugated polymer materials, the following factors need to be considered:

Conductivity: The conductivity of conjugated polymer materials is closely related to their conjugated structure. Conjugate structure refers to a conjugated chain structure in a molecule that has adjacent unsaturated bonds (such as double bonds or several double bonds). This structure allows electrons to move freely on the molecular chain, thereby achieving good conductivity. Therefore, when selecting conjugated polymer materials, it is necessary to prioritize materials with longer and continuous conjugated structures. By adding dopants, the conductivity of conjugated polymer materials can be improved. Usually, dopants can be inserted into the conjugated chain to form additional electron transport pathways and improve conductivity. Commonly used dopants include organic salts, ionic liquids, and conductive polymers. By selecting appropriate dopants and adjusting their content, the conductivity of conjugated polymer materials can be effectively improved.

Mechanical strength: The resistance trainer needs to have sufficient mechanical strength to withstand the tension and pressure during training. Therefore, it is necessary to choose conjugated polymer materials with higher mechanical strength. The use of composite materials in any application involves mechanical properties. In order to achieve satisfactory mechanical performance for the desired application, a large number of experiments must be conducted (Gayatri et al., 2018).

Durability: Resistance trainers need to withstand long-term use and frequent stretching, so it is necessary to choose conjugated polymer materials with good durability.

Tensile strength: It refers to the maximum stress that a material can withstand under tension, and it measures the ability of a material to resist fracture or failure. Higher tensile strength means that the material is more capable of withstanding tensile forces and has better durability and strength. The tensile strength is mainly attributed to its tempered martensitic structure (Zhang et al., 2019). The formula for calculating tensile strength is as follows:

$$S=\frac{F}{L_{W}}$$ (1)

Among them, S represents the tensile strength, in units of kN/m ; F represents the average tensile strength, in units of N ; $L_{W}$ represents the cross-sectional area of the material sample, in units of mm. The physical properties of common conjugated polymer materials are shown in Table 1.

**TABLE 1:** Physical properties of common conjugated polymer materials.

Material	Tensile strength (MPa)	Mechanical strength (MPa)	Electrical conductivity (S/cm)
PANI	20–30	10–30	10^–3^–10^4^
PTH	40–60	5–20	10^–8^–10^–6^
PAA	50–300	40–150	10^3^–10^7^

Common conjugated polymer materials include polyaniline (PANI), polythiophene (PTH), and polyacetylene (PAA). Among them, polyaniline has high conductivity and mechanical strength, but its durability is poor; polythiophene has good durability, but its mechanical strength is low; polyacetylene has good mechanical strength and conductivity. Therefore, this article selects polyacetylene as the material for the resistance training device. When blending conjugated polymer materials, the conductivity and mechanical properties of the materials can be controlled by doping other materials or adjusting the conjugated structure. For example, nanomaterials such as carbon nanotubes can be doped to improve the conductivity of conjugated polymer materials. The mechanical properties of conjugated polymer materials can be adjusted by introducing different functional groups into the conjugated structure. Conjugated polymers have unique advantages such as low cost, high chemical stability, and molecular tunable optoelectronic properties (Dai and Liu, 2020).

(2) Design ideas and device structure

When designing the device structure of a resistance trainer, the following factors need to be considered:

Mechanical properties: The device structure needs to have sufficient mechanical strength to withstand the tension and pressure during training. In addition, the device structure also needs to have a certain degree of elasticity to ensure the safety and comfort of the trainer.

Comfort of use: The structure of the device needs to comply with ergonomic principles to ensure the comfort and stability of the trainer during use. When designing resistance bands, it is necessary to consider their length, width, and material softness to ensure that they can fit properly to various parts of the body and maintain stability during training.

Convenience of operation: The device structure needs to be designed to be simple and easy to use, in order to facilitate the trainer’s adjustment and control of training intensity during use. When designing resistance devices, adjustable resistance intensity devices can be used, allowing trainers to adjust according to their own needs.

According to different training needs, different forms of instrument structures can be designed. Resistance bands can be used for whole body muscle training, using different postures and tension levels to train muscles in different parts. Resistance devices can be designed for targeted muscle training, such as shoulders, arms, chest, *etc.* When designing resistance bands, it is possible to consider using bands of different materials and strengths to meet the needs of different training stages and trainers. When designing resistance devices, adjustable springs and other devices can be used to achieve adjustment of different resistance strengths. At the same time, in order to increase the diversity of training, multiple resistance devices with different resistance intensities can also be designed, so that the trainer can make adjustments as needed.

(3) Preparation method and process flow

Material allocation: Polyacetylene is used as the material for resistance training devices, and carbon fibers are added to increase the volume and strength of the material, changing its resistance characteristics. Adding antioxidants is used to improve the thermal stability and weather resistance of polyacetylene materials, preventing their degradation or fading during processing and use. Polyacetylene, carbon fiber, and antioxidants are mixed and formulated in the proportions of 80%, 18%, and 2%.

Material pretreatment: By mechanical stirring, raw materials such as polyacetylene, antioxidants, and carbon fibers are mixed and refined. Filters are used to filter the mixed material to remove impurities and particles. Vacuum drying is chosen to dry the mixed and filtered materials to remove moisture. Humidity may have a negative impact on the performance and processing of some materials, so ensuring material dryness is crucial.

Forming: The pre processed material is fed into the extruder and extruded from the outlet of the mold through heating and pressure, forming a continuous cross-sectional shape.

Heat treatment: Polyacetylene is a conjugated polymer material with an amorphous structure. To undergo crystallization, first, polyacetylene is heated to a temperature between 60°C and 100°C to make it soft and stretchable. Then, by slowly cooling or soaking it in an appropriate solvent, polyacetylene is formed into an ordered crystalline structure, which helps to improve its crystallinity and mechanical strength.

Polyacetylene improves its mechanical properties through thermal curing. During this process, the polyacetylene sample is processed by extrusion and heated to between 150°C and 200°C to promote cross-linking reaction and form a three-dimensional network structure, which can improve the strength, hardness, and heat resistance of polyacetylene.

**FIGURE 1:**
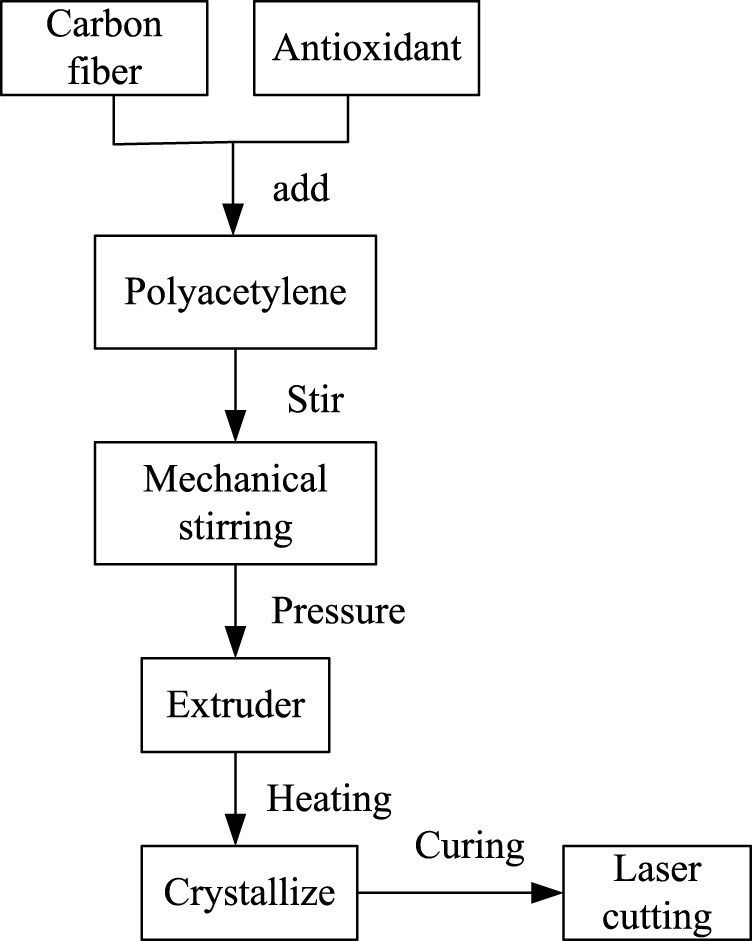
Preparation process of resistance trainer.

Post processing: Laser cutting is used to cut polyacetylene products into the desired shape and size. Cutting tools, such as cutting machines, wire cutters, laser cutting, *etc.*, can be used to complete this step. Sandpaper is used to level and trim the edges and surfaces of polyacetylene products. Polishing agents are used to polish polyacetylene products, resulting in better surface gloss and smoothness. The preparation process of the resistance trainer is shown in Figure 1.

## 3 Strain rate of polyacetylene materials

The change in strain rate can induce the transformation amount and rate of martensite, as well as the differences in the transformation amount and rate of slip lines, dislocations, stacking faults, and deformation twin density in the internal structure (Liu et al., 2019). The material undergoes elastic-plastic deformation and exhibits a uniaxial stress-strain relationship at room temperature (Zheng et al., 2018). Conjugated polymer materials typically have nonlinear stress-strain relationships, and their dynamic mechanical properties are greatly affected by strain rates (Abdullah, 2022). Therefore, the strain rate function is used to describe the strain rate dependence of conjugated polymer materials. In the design and use of resistance trainers, the use of strain rate functions can better understand the mechanical response characteristics of materials (Zeynep, 2020). By controlling the strain rate, the resistance level provided by the trainer can be adjusted and the stimulation effect on muscle groups can be altered. The mechanical parameters of polyacetylene materials under different strain rates are shown in Table 2.

**TABLE 2:** Mechanical parameters of polyacetylene materials under different strain rates.

Strain rate (s^-1^)	Yield strength (MPa)	Tensile strength (MPa)	Modulus of elasticity (GPa)
$3.33\times 10^{-3}$	35.7	53.2	1.25
$6.67\times 10^{-2}$	33.1	44.6	1.12
$1.33\times 10^{-1}$	31.2	39.3	1.05
3.33	12.8	19.3	0.46

The compressive stress-strain relationship of polyacetylene materials can be expressed as:

$$\sigma_{c}\left(\varepsilon \right)=f\left(\varepsilon \right)M\left(\varepsilon ,\dot{\varepsilon }\right)$$ (2)

In the formula: $f\left(\varepsilon \right)$ is the stress-strain function under quasi-static state; $M\left(\varepsilon ,\dot{\varepsilon }\right)$ is the strain rate function, taking the following formula:

$$\left\{\begin{array}{c} M\left(\varepsilon ,\dot{\varepsilon }\right)={\left(\dot{\varepsilon }/{\dot{\varepsilon }}_{0}\right)}^{n\left(\varepsilon \right)}\\ n\left(\varepsilon \right)=a+b\varepsilon \end{array}\right.$$ (3)

Among them, $n\left(\varepsilon \right)$ is the exponential function of strain rate; ${\dot{\varepsilon }}_{0}$ is the quasi-static strain rate, and a,b are constants.

Taking the apparent density of polyacetylene material as an example, its a,b can be obtained by the following method.

$$M\left(\varepsilon ,\dot{\varepsilon }\right)=\sigma_{c}\left(\varepsilon \right)/f\left(\varepsilon \right)=\sigma_{c}\left(\varepsilon \right)/{\left[\sigma_{c}\left(\varepsilon \right)\right]}_{{\dot{\varepsilon }}_{0}=3.33\times 10^{-3}}$$ (4)

In the formula, ${\left[\sigma_{c}\left(\varepsilon \right)\right]}_{{\dot{\varepsilon }}_{0}=3.33\times 10^{-3}}$ is the compressive stress MPa when ${\dot{\varepsilon }}_{0}=3.33\times 10^{-3}$ $s^{-1}$ .

For a given compressive strain of ε , the strain rate exponential function $n\left(\varepsilon \right)$ can be obtained:

$$n\left(\varepsilon \right)=\frac{\ln M\left(\varepsilon ,\dot{\varepsilon }\right)}{\ln\left(\varepsilon /\varepsilon_{0}\right)}$$ (5)

In the formula, $\varepsilon_{0}$ is the compressive strain under static load conditions.

In order to provide the relationship between the static and dynamic mechanical parameters of polyacetylene materials from the perspective of engineering approximation analysis, fitting regression is conducted considering the apparent density, and the following regression formula is obtained:

$$\sigma =A\rho^{B}$$ (6)

In the formula, A,B are regression coefficients.

## 4 Resistance trainer physical strength tensile training test

This study selected 12 participants for testing experiments in a fitness center and divided them into an experimental group and a control group. The experimental group used resistance trainers made of conjugated polymer materials, while the control group used traditional resistance trainers. The selection criteria for each group of testers are shown in Table 3.

**TABLE 3:** Selection criteria for testers.

Tester	Gender	Age	Weight (kg)	Height (cm)	Whether to exercise regularly
1	Male	22 ± 1.5	55 ± 1.5	170 ± 2	Beginner
2	Male	25 ± 1.5	60 ± 1.5	175 ± 2	Have a certain training foundation
3	Male	25 ± 1.5	65 ± 1.5	180 ± 2	Experienced training
4	Female	22 ± 1.5	50 ± 1.5	165 ± 2	Beginner
5	Female	25 ± 1.5	55 ± 1.5	170 ± 2	Have a certain training foundation
6	Female	25 ± 1.5	58 ± 1.5	175 ± 2	Experienced training

(1) Resistance adjustment performance test

**FIGURE 2:**
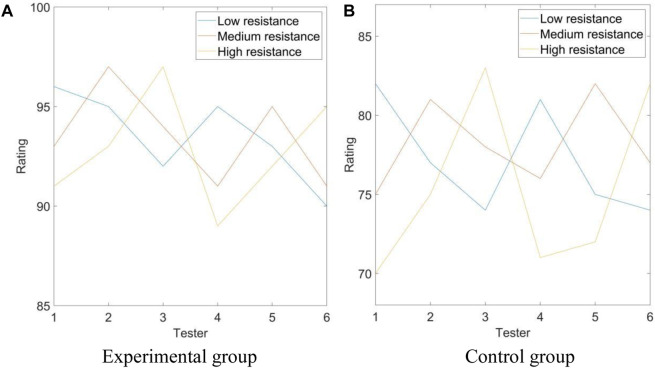
Resistance adjustment performance. **(A)** Experimental group **(B)** Control group.

Experimental steps: Based on the needs and ability range of the trainers, this study set three levels of low resistance, medium resistance, and high resistance, and required each tester to conduct a tensile test. For different resistance levels, they were rated based on test performance results, with a maximum score of 100 points. The score was evaluated based on the tensile strength and technical level of the tester to accurately reflect their training effectiveness at various resistance levels. The experimental results are shown in Figure 2.

In Figure 2, the *x*-axis represents the tester, and the *y*-axis represents the score. The legends show low resistance, medium resistance, and high resistance, respectively.

As shown in Figure 2A, it can be seen that when the resistance was set to low in the experimental group, the highest score for the performance of the test personnel was 96 points, and the lowest score was 90 points. When the experimental group set the resistance to medium resistance, the highest score for the performance of the tester was 97 points, and the lowest was 91 points. When the experimental group set resistance to high resistance, the highest score for the performance of the tester was 97 points, and the lowest score was 89 points. As shown in Figure 2B, it can be seen that when the resistance was set to low in the control group, the highest score for the performance results of the test personnel was 82 points, and the lowest score was 74 points. When setting resistance as medium in the control group, the highest score for the performance of the test personnel was 82 points, and the lowest score was 75 points. When the resistance was set to high in the control group, the highest score for the performance of the test personnel was 83 points, and the lowest score was 70 points. As shown in Figure 2, the performance scores of the test personnel in the experimental group were higher than those in the control group.

(2) Rebound performance test

Experimental purpose: By using standard tensile training movements, the rebound performance of conjugated polymer material resistance trainers was compared with traditional resistance trainers. The advantages of the conjugated polymer material resistance trainer in improving training stability and efficiency were evaluated by measuring its speed and degree of recovery after tensile release (Deng et al., 2022).

**FIGURE 3:**
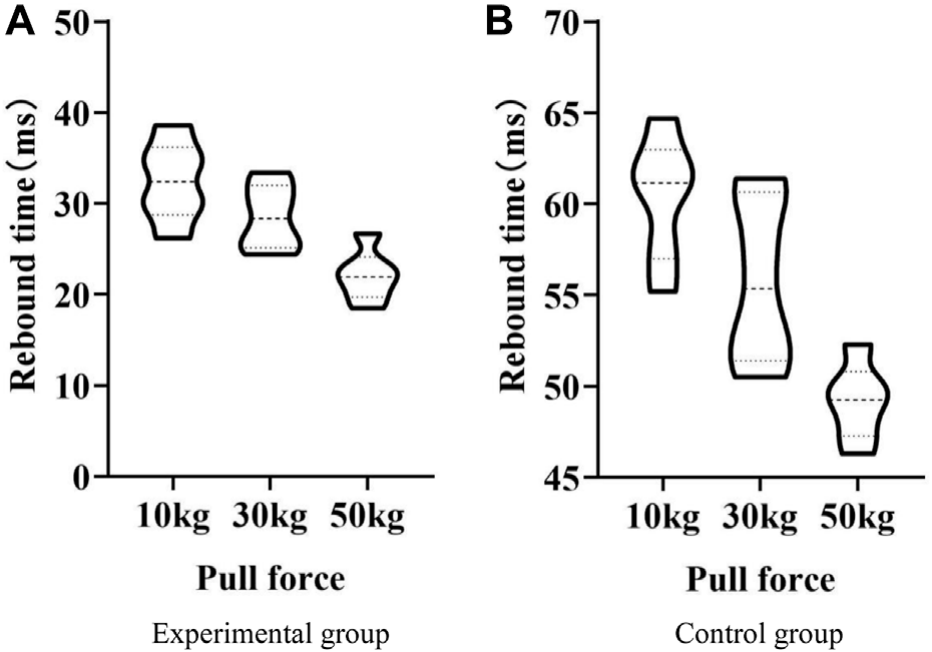
Rebound performance. **(A)** Experimental group **(B)** Control group.

Experimental steps: Firstly, the ruler measured the initial length of the trainer when it was used without tension. Forces of 10kg, 30kg, and 50 kg were used, and tensile forces were applied to conjugated polymer material resistance trainers and traditional trainers (Zeng et al., 2018). After the tension was released, the speed and degree to which each trainer recovers to its initial length were observed and recorded. The experimental results are shown in Figure 3 and Table 4.

**TABLE 4:** Change in rebound length.

Tensile force (kg)	Resistance trainer in the paper		Traditional resistance trainer	
	Initial length (cm)	Length after recovery (cm)	Initial length (cm)	Length after recovery (cm)
10	150	150.2	150	152.4
30	150	150.9	150	153.7
50	150	151.6	150	155.3

(3) Durability testing

In Figure 3, the *x*-axis represents the tensile force, which is 10kg, 30kg, and 50 kg respectively; the *y*-axis represents the rebound time (ms).

As shown in Figure 3A, when the tensile force was applied to 10 kg in the experimental group, the rebound time was between 26 ms and 39 ms. When the tensile force was applied to 30 kg in the experimental group, the rebound time was between 24 ms and 34 ms. When the tensile force was applied to 50 kg in the experimental group, the rebound time was between 18 ms and 27 ms. As shown in Figure 3B, the rebound time in the control group was between 55 ms and 65 ms when the tensile force was applied to 10 kg. When the tensile force was applied to 30 kg in the control group, the rebound time was between 50 ms and 62 ms. The rebound time in the control group was between 45 ms and 53 ms when the tensile force was applied to 50 kg. As shown in Figure 3, the required rebound time of the experimental group was lower than that of the control group, indicating that the conjugated polymer material resistance trainer has better elasticity.

Experimental purpose: The durability performance of the conjugated polymer material resistance trainer was evaluated, including strength, wear resistance, and temperature resistance tests.

Experimental steps:

Strength testing: By applying gradually increasing pressure, the strength and durability of the material were tested to determine its deformation and failure performance under different stresses.

Wear resistance test: Through a wear testing machine, the surface of the material was subjected to wear testing, and the friction and wear conditions in actual use were simulated to evaluate the wear resistance and service life of the material.

**FIGURE 4:**
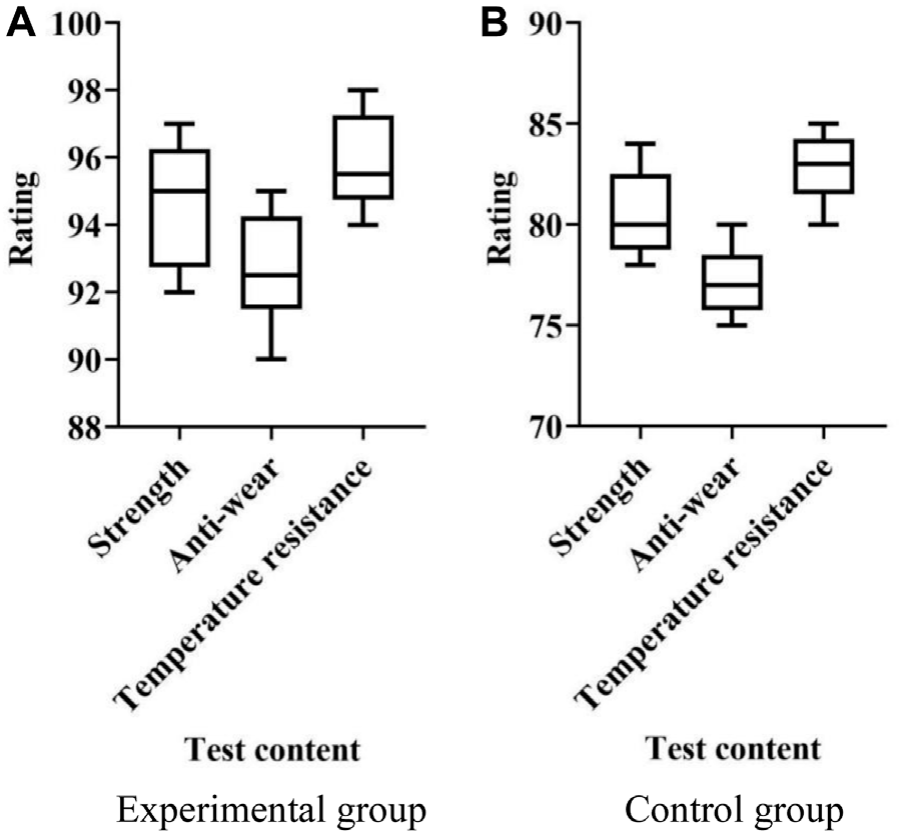
Durability. **(A)** Experimental group **(B)** Control group.

Temperature resistance test: The material was placed in an environment with periodic temperature changes to evaluate its physical properties and durability under temperature changes. This can simulate the temperature changes encountered by materials in actual use, and check their thermal expansion, shrinkage, thermal aging and other characteristics. Based on the observed experimental results, it is scored with a maximum score of 100 points. The experimental results are shown in Figure 4.

In Figure 4, the *x*-axis represents the testing content, which includes strength, wear resistance, and temperature resistance tests; the *y*-axis represents the score.

As shown in Figure 4A, the strength test scores in the experimental group ranged from 92 to 97 points. In the experimental group, the scores for wear resistance testing ranged from 90 to 95 points. The temperature tolerance test scores in the experimental group ranged from 94 to 98. As shown in Figure 4B, the strength test scores in the control group ranged from 78 to 84 points. In the control group, the score of wear resistance test was between 75 and 80 points. The temperature tolerance test score in the control group ranged from 80 to 85 points. As shown in Figure 4, the scores of the experimental group were higher than those of the control group, indicating that the yoke polymer material resistance trainer has better durability.

(4) Training effectiveness evaluation

**FIGURE 5:**
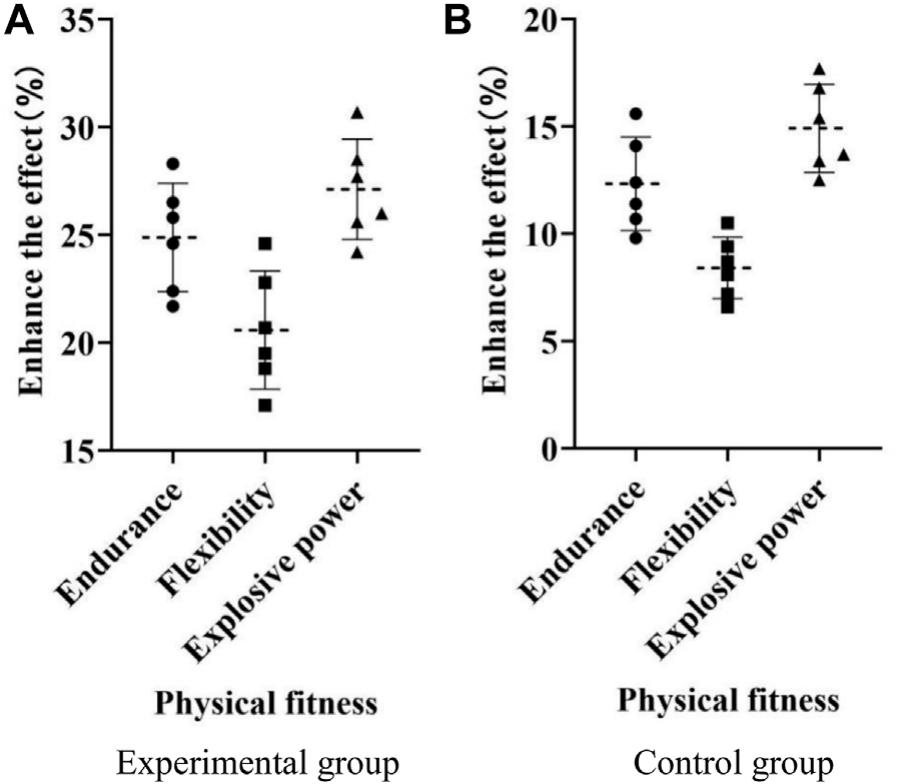
Training effect. **(A)** Experimental group **(B)** Control group.

This study conducted the same physical fitness training for all testers, lasting for 3 months. The improvement effects of each tester’s physical fitness, including endurance, flexibility, and explosive power, were recorded. The experimental results are shown in Figure 5.

In Figure 5, the *x*-axis represents physical fitness, which includes endurance, flexibility, and explosive power; the *y*-axis represents the improvement effect (%).

As shown in Figure 5A, after 3 months of training, the testers in the experimental group improved their endurance by 21%–29%; the testers in the experimental group improved their flexibility by 17%–25%; the testers in the experimental group increased their explosive power by 24%–31%. As shown in Figure 5B, after 3 months of training, the testers in the control group improved their endurance by 9%–16%; the testers in the control group improved their flexibility by between 6% and 11%; the testers in the control group increased their explosive power by 12%–18%. As shown in Figure 5, the improvement effect of the experimental group after 3 months of training was higher than that of the control group.

## 5 Conclusion

This article took conjugated polymer materials as the research object, and explored their application effects and advantages in the field of resistance training by analyzing and testing their dynamic mechanical properties. Conjugated polymer materials have broad application potential and can be prepared into various forms and types of resistance devices in the field of resistance training, providing diverse and personalized choices for daily physical strength tensile training. Its plasticity and adjustability are not possessed by traditional materials, and resistance devices of different shapes, sizes, and strengths can be prepared according to the needs and purposes of trainers. Compared with traditional materials, conjugated polymer materials have better tensile properties and durability, can withstand greater tensile and extrusion forces during resistance training, and are less prone to problems such as fatigue and damage. The experimental results showed that the resistance trainer made of conjugated polymer materials could improve the training effect and user experience. When using resistance bands made of polyacetylene for muscle stretching training, the trainer can freely adjust the size of the tension as needed to achieve different intensity training. The research results indicated that conjugated polymer materials have good plasticity and adjustability, and have important advantages in the preparation of resistance devices. At the same time, conjugated polymer materials exhibit excellent tensile performance and durability in resistance training, which can effectively meet the needs and requirements of trainers. Due to time constraints, this paper has provided a brief introduction to the preparation and performance testing of conjugated polymer materials, but has not provided a detailed discussion on their optimization schemes and methods for improving performance, lacking in-depth technical exploration. In the future, intelligent technology can be combined to develop resistance trainers for the dynamic mechanical properties of conjugated polymer materials, achieving real-time monitoring and analysis of training data, and providing trainers with more personalized training plans and better training effects.

## Data Availability

The original contributions presented in the study are included in the article/Supplementary Material, further inquiries can be directed to the corresponding author.
